# Transient Receptor Potential Vanilloid1 (TRPV1) Channel Opens Sesame of T Cell Responses and T Cell-Mediated Inflammatory Diseases

**DOI:** 10.3389/fimmu.2022.870952

**Published:** 2022-05-11

**Authors:** Tengfei Xiao, Mingzhong Sun, Jingjing Kang, Chuanxiang Zhao

**Affiliations:** ^1^ Department of Clinical Laboratory, The Sixth Affiliated Hospital of Nantong University, Yancheng Third People’s Hospital, Yancheng, China; ^2^ Department of Clinical Laboratory, Affiliated Hospital of Nanjing University Medical School, Yancheng First People’s Hospital, Yancheng, China; ^3^ Institute of Medical Genetics and Reproductive Immunity, School of Medical Science and Laboratory Medicine, Jiangsu College of Nursing, Huai’an, China

**Keywords:** TRPV1, T cell, Ca2+, fever, T cell-mediated inflammatory diseases

## Abstract

Transient receptor potential vanilloid1 (TRPV1) was primarily expressed in sensory neurons, and could be activated by various physical and chemical factors, resulting in the flow of extracellular Ca^2+^ into cells. Accumulating data suggest that the TRPV1 is expressed in some immune cells and is a novel regulator of the immune system. In this review, we highlight the structure and biological features of TRPV1 channel. We also summarize recent findings on its role in modulating T cell activation and differentiation as well as its protective effect in T cell-mediated inflammatory diseases and potential mechanisms.

## Introduction

Transient receptor potential (TRP) is a large superfamily of nonselective cation channels comprising of 28 members mainly located on the cell membrane. The TRP superfamily can be divided into TRPC (Canonical/Classical), TRPV (Vanilloid) and TRPM (Melastatin) sub-families (1). TRPV sub-families can be activated by vanillic acid compounds consisting of TRPV 1-6 (2). In 1997, TRPV1 was identified as a receptor of capsaicin, the main pungent component in “hot” chilli pepper (3). Over the past few decades, TRPV1 has been widely studied in the nervous system. In the peripheral nervous system, TRPV1 channel was found to be highly expressed in the spinal dorsal root ganglion neurons, the trigeminal ganglion and primary sensory neurons, which mainly mediate pain perception, transmission and regulation process. In the central nervous system, the TRPV channel was mainly involved in the regulation of body temperature, release of synaptic neurotransmitters, synaptic transmission and apoptosis (4). In addition, recent studies have revealed that TRPV1 was widely expressed in non-neuronal cell membranes of the kidney, pancreas, testes, uterus, spleen, stomach, small intestine, lung and liver mucous gland (2). Besides, the TRPV1 channel has been shown to play an important role in the immune system.

In this review, we discuss the structure and biological characteristics of the TRPV1 channel and highlight ecent findings on the roles of the TRPV1 channel in controlling T cell activation and differentiation. We also discuss the protective functions of the TRPV1 in T cell-mediated inflammatory diseases and the underlying potential mechanisms.

## The Structure and Biological Characteristics of the TRPV1 Channel

**Figure 1:**
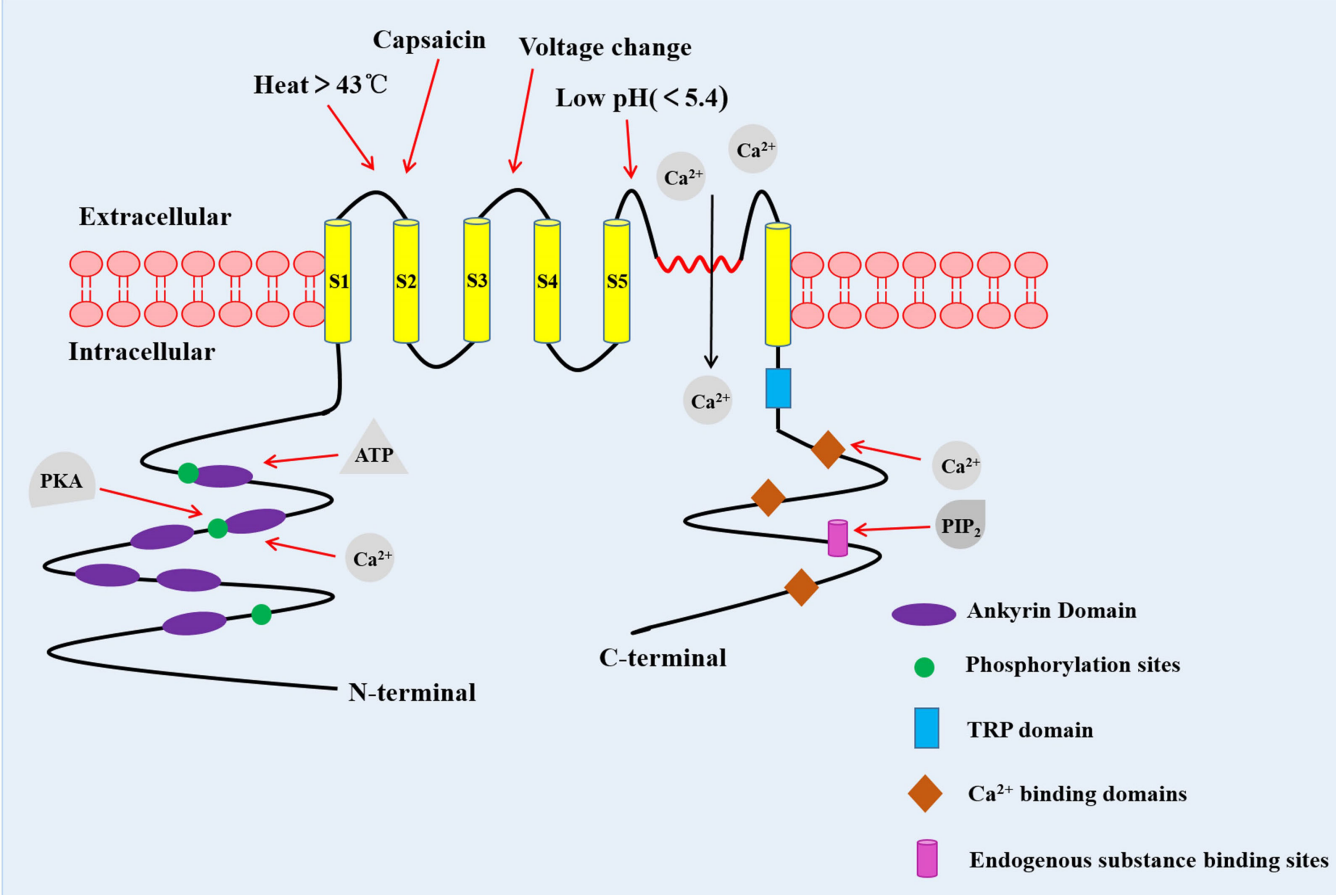
The structure of the TRPV1 channel. TRPV1 channel is a homologous tetramer composed of four subunits, with six-transmembrane domains and a pore-forming hydrophobic group between the fifth and sixth transmembrane domains. The N-terminal contains several phosphorylation sites and six ankyrin repeat domains. The C-terminal has a TRP domain, multiple calmodulin blinding domains and binding sites of endogenous substance. PKA, Protein kinase A (10).

TRPV1 channel is a coding protein with a molecular weight of 95 kDa, composed of 838 amino acids. Sequence analysis data has shown that the TRPV1 channel is a homologous tetramer composed of four subunits, each of which has six-transmembrane domains with a pore-forming hydrophobic group between the fifth and sixth transmembrane domains (5). Its N-terminal and C-terminal regions are located in the inner side of the cell membrane to regulate the receptor functions. The N-terminal contains several phosphorylation sites and six ankyrin repeat domains, which bind calmodulin and ATP and modulate the sensitivity and functions of the TRPV1 (6, 7). On the other hand, the C-terminal bears a TRP domain, multiple calmodulin binding domains and endogenous substance binding sites, such as phosphatidyl-inositol-4,5-bisphosphate (PIP_2_) (8, 9) (**Figure 1**).

The TRPV1 is a multimodal receptor, which is activated and/or allosterically modulated by a range of thermal, mechanical and chemical stimuli (11). Besides capsaicin, TRPV1 channel is also activated by a variety of other plant-derived vanilloids, including camphor and resiniferatoxin (RTX), and putative endogenous vanilloids such as the endocannabinoid, inflammatory mediators such as arachidonic acid (12, 13). The thermal sensitivity of the TRPV1 was shown to be enhanced by various pro-inflammatory factors, such as nerve growth factor (NGF), bradykinin, lipid, prostaglandin and ATP (14). Although many studies have evaluated the role of PIP_2_ in the activation of TRPV1, the data still remains controversial. For instance, Yao et al. demonstrated that PIP_2_ could fuel the activation of TRPV1 (15, 16), while other studies reported that PIP_2_ inhibited the TRPV1 activation (17, 18). Since the membrane is a highly asymmetric lipid bilayer, the contradictory effects of PIP_2_ on the TRPV1 may be depending on which leaflet of the cell membrane it interacts with. Insertion of the PIP_2_ into the inner leaflet of the plasma membrane enhanced the response of capsaicin in activating the TRPV1, while insertion into both leaflets suppressed the channel activation (19). Other activators of the TRPV1 channel include heat (>43 °C), low pH (< 5.4), static charge and voltage change (13). It has been demonstrated that TRPV1 is intrinsically heat sensitive (18), and temperature sensing is associated with voltage-dependent gating in the heat-sensitive channel TRPV1 (20).

After the TRPV1 activation, extracellular Ca^2+^ flows into the cells, and the intracellular the Ca^2+^ pool releases, resulting in increased concentration of intracellular Ca^2+^ (21). This increased intracellular Ca^2+^ mediates the basic activities of many cells, such as muscle contraction, neuronal activity, transmitter release, cell proliferation and apoptosis. In addition, activated TRPV1 can regulate body temperature and pain (22, 23).

## The Role of the TRPV1 Channel in T Cell Responses

### Functional Expression and TCR-Mediated Activation of TRPV1 in CD4^+^ T Cells

Some previous studies analyzed the expression of TRPV1 mRNA and protein in human peripheral blood mononuclear cells (PBMC) (24), and found that they were expressed in mouse and rat thymocytes (25, 26). Thereafter, other studies demonstrated the expression of TRPV1 on human NK and CD3^+^ T cells (27, 28), as well as in primary mouse and human T cells and human T cell line (Jurkat cells) (24, 28–32). Thus, the TRPV1 channel might play a pivotal role in T cells.

**Figure 2:**
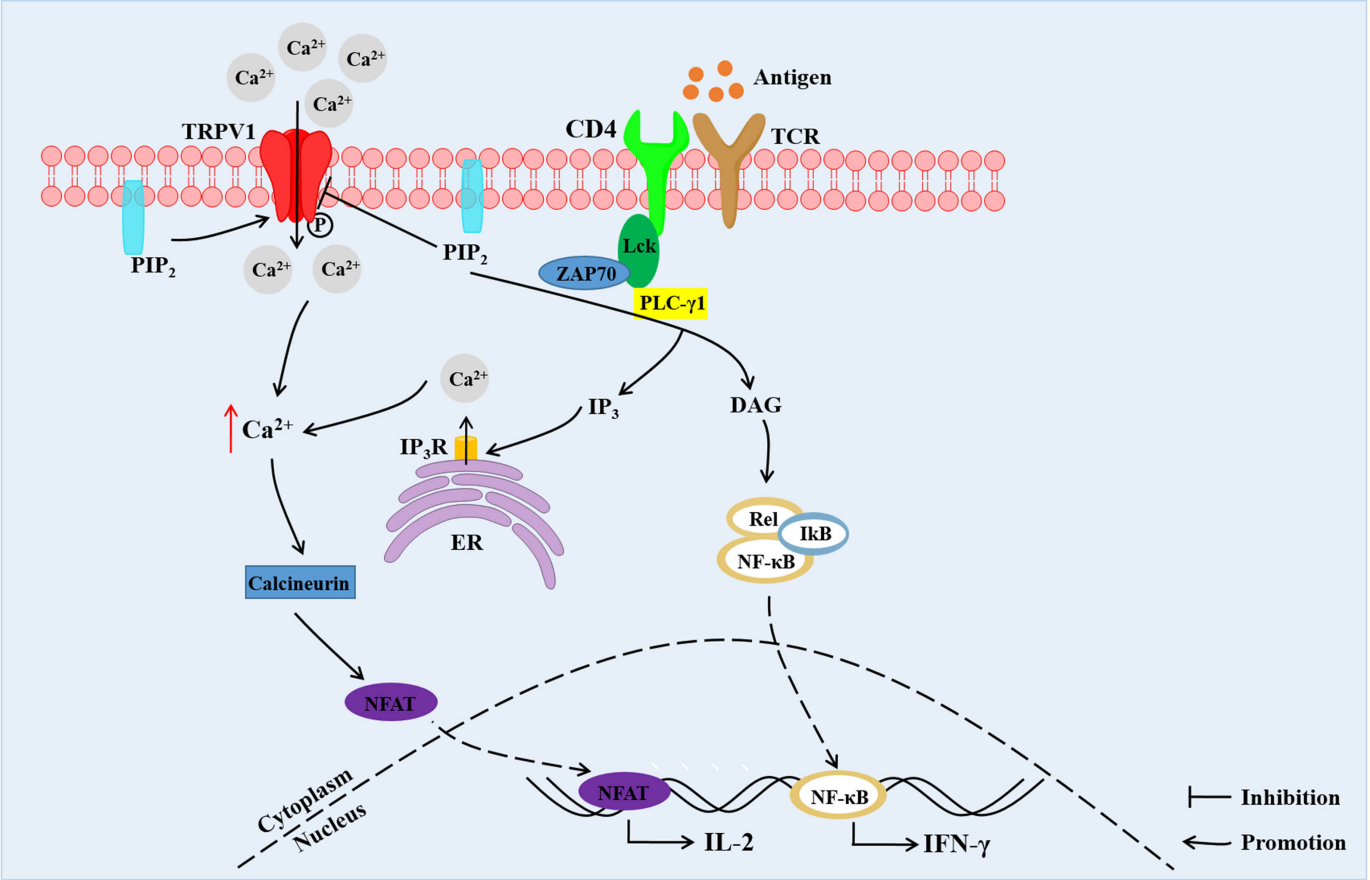
The TCR signals and TRPV1-mediated increase in Ca^2+^ concentration and downstream Ca^2+^-dependent signaling in CD4^+^ T cells. TRPV1 is bound with CD4 and Lck. TRPV1 mediates Ca^2+^ influx, and the tyrosine of TRPV1 is phosphorylated by Lck. PIP_2_ located in both leaflets suppresses the activation of TRPV1. Hydrolysis of PIP_2_ into DAG and IP_3_ by PLC-γ1 leads to relieving of PIP_2_-mediated inhibition of TRPV1. Besides, IP_3_ binds to IP_3_R on ER contributing to the release of Ca^2+^ from intracellular Ca^2+^ store. The increased Ca^2+^ concentration promotes migration of NFAT into the nucleus, inducing the expression of IL-2. DAG promotes the entry of NF-κB into the nucleus, resulting in IFN-γ expression. PIP_2_, phosphatidylinositol-4,5-bisphosphate; PLC-γ1, phospholipase C gamma 1; IP_3_, inositol 1,4,5-trisphosphate; DAG, diacylglycerol; IP_3_R, IP_3_ receptor; ER, endoplasmic reticulum; NFAT, nuclear factor of activated T-cells; NF-κB, nuclear factor kappa binding (33).

The activation and function of TRPV1 could be modulated by TCR-induced signaling pathway. In resting and TCR-stimulated CD4^+^ T cells, TRPV1 binds TCR co-receptor CD4 and Src-family tyrosine kinase Lck (33). The tyrosine of TRPV1 was rapidly phosphorylated by Lck in response to TCR stimulation leading to inactivation of TRPV1, which was not modified in Lck-deficient T cells (33). In addition, PIP_2_ in the intracellular leaflet of the plasma membrane was shown to activate TRPV1. In contrast, PIP_2_ located in both leaflets suppressed the activation of the TRPV1 (19). PIP_2_ was hydrolyzed into diacylglycerol (DAG) and inositol 1,4,5-trisphosphate (IP_3_) by TCR-induced activated phospholipase C gamma 1 (PLC-γ1) (33). The hydrolysis of PIP_2_ relieved the PIP_2_-mediated inhibition of the TRPV1 (16). Besides, IP_3_ binds to its receptor (IP_3_R) on endoplasmic reticulum (ER), contributing to the release of Ca^2+^ from the intracellular Ca^2+^ pool (33) (**Figure 2**).

### The TCR Signals and TRPV1 Increase Ca^2+^ in CD4^+^ T Cells

The elevation of intracellular Ca^2+^ is required for T cell activation, proliferation, differentiation and effector functions (34). The engagement of TCR increases the intracellular Ca^2+^ concentration, which results from a dual Ca^2+^ response; Ca^2+^ release from the ER stores and Ca^2+^ influx from the extracellular milieu into the cytosol across the plasma membrane (34). This in turn leads to activation of downstream Ca^2+^-dependent signaling pathways and nuclear translocation of key transcription factors, which include nuclear factors of activated T-cells (NFAT) and nuclear factor kappa binding (NF-κB) (35). These activities account for T cell responses such as production of various cytokines, as well as proliferation and differentiation into effector cells.

TRPV1 functions as Ca^2+^-permeable channels on the T cell plasma membrane. For instance, a previous study showed that Capsaicin, a special TRPV1 channel agonist, increased Ca^2+^ influx and intracellular Ca^2+^ concentration in activated CD4^+^ T cells, but did not affect resting T cells (36, 37). TRPA1 inhibited the TRPV1 channel activity while deletion of TRPA1 in CD4^+^ T cells increased T-cell receptor-induced Ca^2+^ influx (38). Besides, TRPV1 protein deficiency in CD4^+^ T cells reduced activation of NFAT and NF-κB in response to TCR stimulation and decreased secretion of IL-2 and IFN-γ (31). Moreover, TRPV1 increased Ca^2+^ influx upon stimulation of phytohemagglutinin (PHA) (39). On the contrary, TRPV1-mediated Ca^2+^ influx was not influenced by ionomycin (a Ca^2+^ ionophore) and thapsigargin (a sarcoplasmic reticulum Ca^2+^-ATPase pump inhibitor), which is known to mediate TCR-independent Ca^2+^ activation (31). These studies demonstrated that TRPV1 is a non-store-operated Ca^2+^ channel which modulates TCR-induced Ca^2+^ influx in T cells (31) (**Figure 2**).

TRPV1 not only promotes T cell activation, but induces T cell death. Previous studies demonstrated that apoptosis of human peripheral T and Jurkat cells were induced in response to exposure to prolonged and high capsaicin concentration (25, 37). Besides, capsaicin-induced apoptosis was associated with intracellular free Ca^2+^ influx (37). In addition, treatment of thymocytes with capsaicin induced autophagy through ROS-regulated AMPK and Atg4C pathways (26). However, the ROS generation was not associated with Ca^2+^ signaling (37).

### Temperature Changes Determine the Fate of CD4^+^ T Cells *via* TRPV1

Similar to free Ca^2+^, temperature changes have been shown to activate the immune system (40). Fever is a physiological response to infections, injuries and inflammation. Fever-range temperatures (1°C∼4°C above basal body temperature) are rapidly induced in response to an infection, which in turn boosts protective immune responses, such as immune surveillance. Two studies showed that fever-range temperatures (38∼41°C) could promote lymphocytes homing to secondary lymphoid tissues through enhancement of L-selectin and α4β7 integrin-dependent adhesive interactions between circulating lymphocytes and specialized high endothelial venules, thus increasing immune surveillance (41, 42). Another study revealed that fever promoted trafficking of T cells and enhanced immune surveillance during an infection through heat shock protein 90 (HSP90)-induced α4-integrin activation and increase of α4-intergrin-mediated T cell adhesion (43). Besides, fever-like whole body hyperthermia (WBH) treatment of mice led to increase in tissue T cells with uropods. Besides, the WBH treatment induced reorganization of protein kinase C (PKC) isozymes and increased PKC activity within T cells (44). In addition, mildly elevated temperature range (≤40°C) was shown to strengthen cytotoxic activities of T cells from both adult and cord blood. However, this phenomenon was attenuated on exposure of the T cells to 42°C for 1 hour (45).

**Figure 3:**
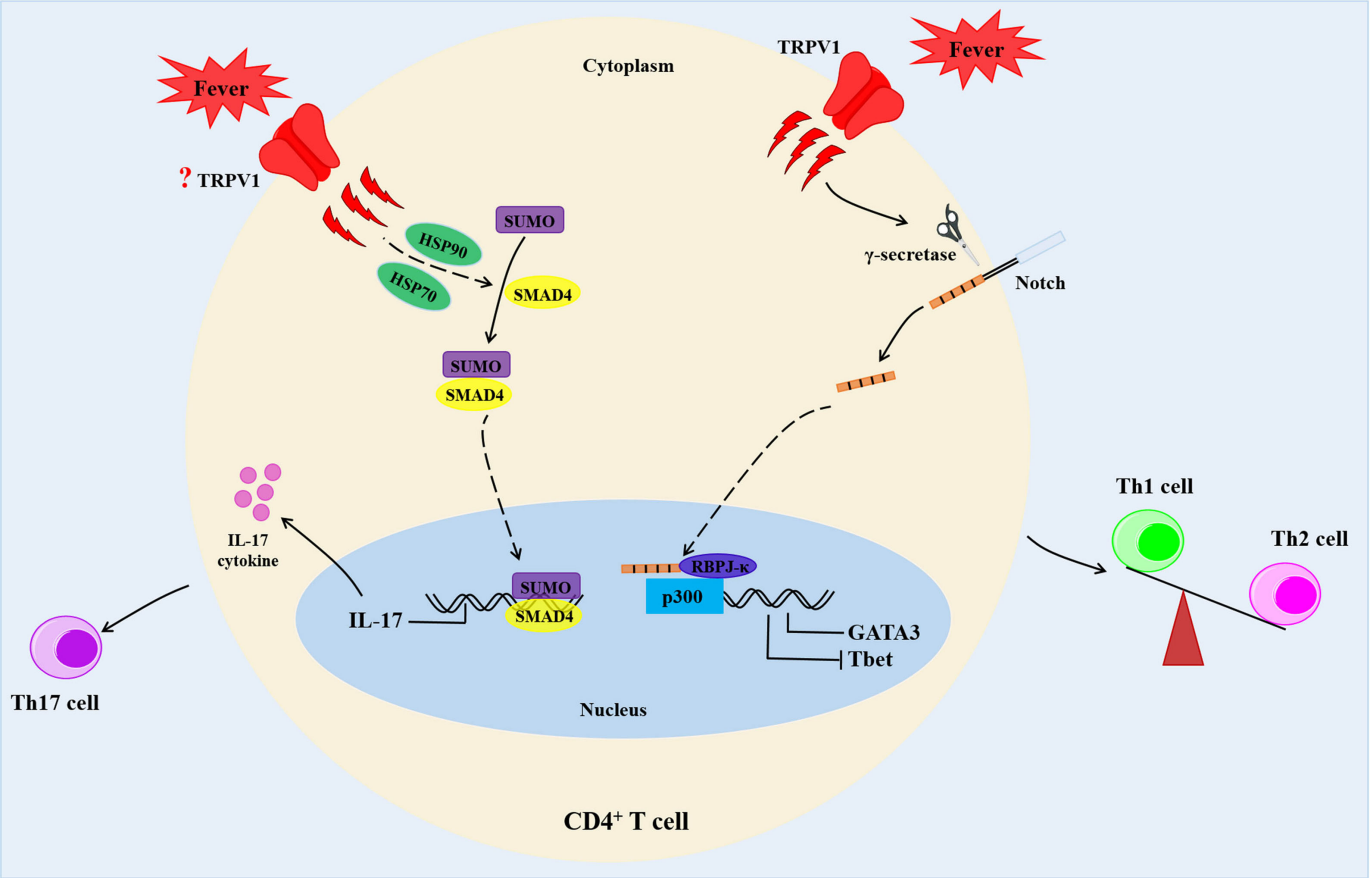
Fever determines the fate of CD4^+^ T cells. Febrile temperature changes enhance Th2 differentiation and reduce Th1 differentiation *via* a TRPV1-regulated Notch-dependent pathway. In addition, febrile temperature promotes Th17 cell differentiation which depends on HSP-70- and HSP-90-related heat shock response and enhances SUMOylation of SMAD4 transcription factor at its K113 and K159 residues. HSP90, heat shock proteins 90; HSP70, heat shock proteins 70.

On the other hand, temperature changes were shown to affect T cell differentiation. Chen Dong et al. reported that febrile temperature did not influence Th1, Th2 and induced Treg (iTreg) cell differentiation, but selectively and robustly promoted Th17 cell differentiation at 39.5°C. Febrile temperature also elevated Th17 cell cytokine genes (IL-17a, IL-17f and IL-22) and reduced the expression of anti-inflammatory cytokine IL-10 (46). Besides, febrile temperature (38.5°C-39.5°C) fueled the pathogenicity of Th17 cells with a highly pro-inflammatory feature and aggravated experimental allergic encephalomyelitis (EAE) model (46). Mechanistically, febrile-temperature-induced Th17 cell differentiation depended on HSP-70- and HSP-90-related heat shock response and enhanced SUMOylation of SMAD4 transcription factor at its K113 and K159 residues, which facilitated its nuclear localization (46). In sync with the previous findings, Gaublomme and colleagues demonstrated that treatment with anti-fever drugs reduced Th17 cell response *in vivo*, while *in vitro* induced Th17 cells were highly pro-inflammatory in a lung-inflammation model (47) (**Figure 3**). In addition, naïve CD8^+^ T cells exposed to 39.5°C *in vitro* promoted the rate of synapse formation with APC, which led to differentiation of a greater percentage of CD8^+^ T cells into effector cells (48). This phenomenon was attributed to an increase in membrane fluidity and clustering of GM1^+^CD-microdomains, as well as clustering of TCRβ and CD8 co-receptor (48). A recent study showed that fever enhanced production of activated CD8^+^ T cell cytokines and glycolytic metabolism with a limited effect on the expression of CD69, the activation marker (49). Moreover, febrile temperature promoted protective antitumor effects of CD8^+^ T cells *via* mitochondrial translation (49). However, data on how the T cells sense subtle temperature changes remain scant.

TRPV1 is a critical regulator of physiological body temperature and fever, outside the central nervous system (50, 51). TRPV1 could be activated at a temperatures threshold near 43°C (52). A previous study demonstrated that fever sensing by CD4^+^ T cells involve TRPV1 channel during CD4^+^ T cell differentiation (53). In addition, fever-range temperatures significantly enhanced Th2 differentiation and reduced Th1 commitment at moderate fever temperature (39°C) *in vitro via* a TRPV1 channel-mediated Notch-dependent pathway. This was accompanied by upregulation of Th2-relevant transcription factor GATA3, and reduction of the Th1-relevant transcription factor, T-bet (53) (**Figure 3**). However, both mouse and human naïve CD4^+^ T cells treatment with temperatures between 37°C and 39°C showed no alterations in the activation, proliferation, or cell survival (53). Samivel R et al. revealed suppression of the production of Th2/Th17 cytokines in CD4^+^ T cells and Jurkat T cells upon genetic and pharmacological inhibition of TRPV1 (32).

Together, these data demonstrated that TRPV1 functions as a temperature sensor in CD4^+^ T cells. The temperature changes could regulate CD4^+^ T cell differentiation through TRPV1.

## The Functions of TRPV1 in T Cell-Mediated Inflammatory Diseases

**Figure 4:**
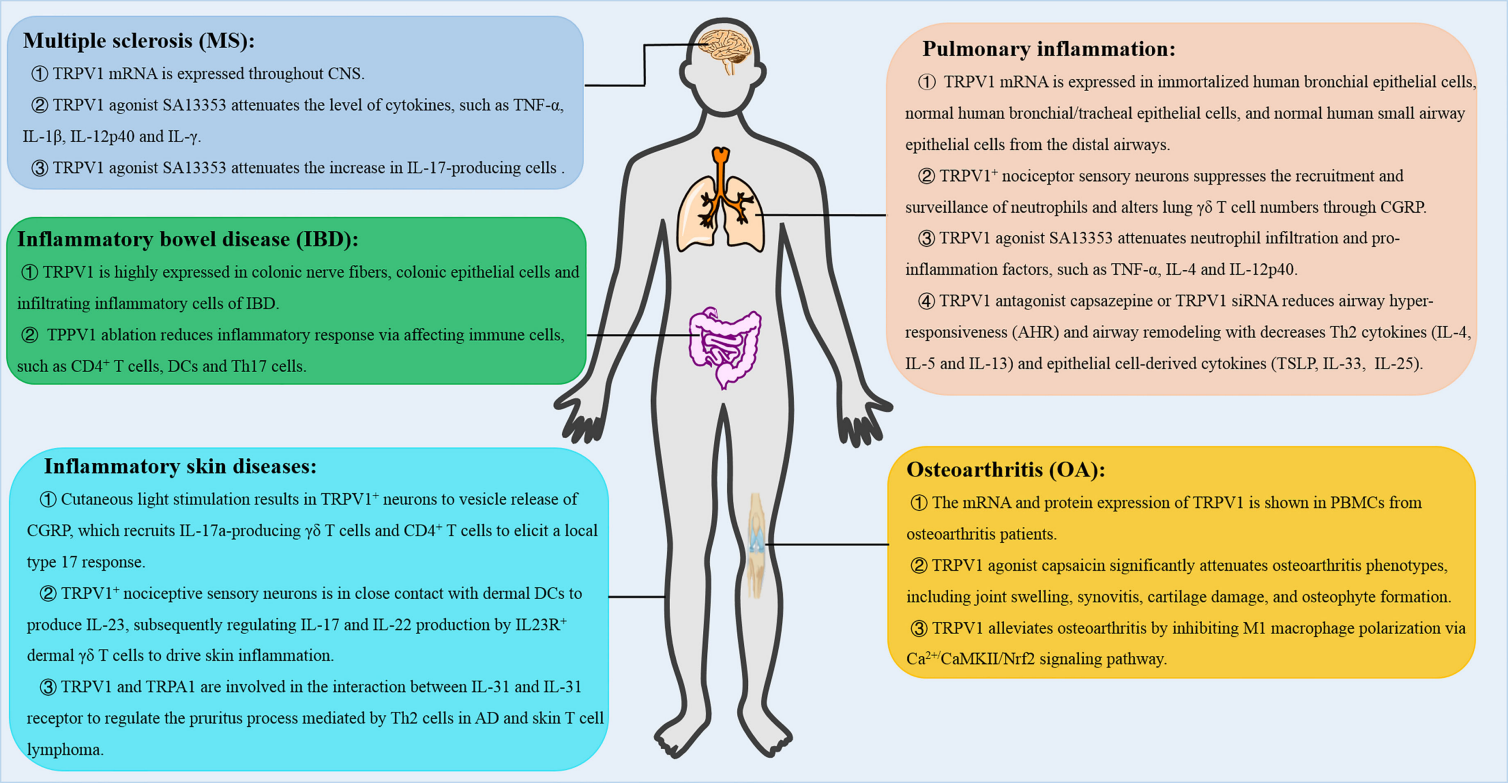
The role of TRPV1 in T cell-mediated inflammatory diseases. TRPV1 regulates the inflammatory responses, such as multiple sclerosis (MS), pulmonary inflammation, inflammatory skin diseases and inflammatory bowel disease (IBD), and osteoarthritis (OA). CNS, central nervous system; AD, atopic dermatitis; CGRP, calcitonin gene-related peptide.

Inflammation is the main and common pathophysiological feature of pain, visceral inflammation, hypertension and cancer at different stages of occurrence and development (54). Inflammation is characterized by redness, swelling, heat, pain, tissue injury or organ dysfunction (54). Inflammation has been shown to remove tissue injuries and promote restoration during immune responses (54). Recent studies have shown that TRPV1 plays anti-inflammatory roles by attenuating acute and chronic inflammatory processes as well as enhancing homeostasis, thus, attenuating harmful effects of inflammatory responses. Here, we analyzed how TRPV1 modulates T cell-mediated inflammatory responses, which include multiple sclerosis (MS), pulmonary inflammation, inflammatory skin diseases or inflammatory bowel diseases (IBD) as well as osteoarthritis (OA) (**Figure 4**).

### Multiple Sclerosis

Multiple sclerosis (MS) is a complex central nervous system autoimmune disease characterized by autoimmune demyelination and neurodegeneration, which are mediated by Th1 and Th17 cells, macrophages, and immune inflammatory mediators. Previously, TRPV1 mRNA was found to be expressed throughout the central nervous system (CNS), but it was highly expressed in sensory neurons of the dorsal root ganglion (10). The TRPV1^+^ neurovascular complex, referred to as the blood-CNS barrier, promoted invasion of pathogenic lymphocytes (55). However, SA13353, a TRPV1 agonist, reduced the number of cytokines, including TNF-α, IL-1β, IL-12p40, IL-17, and interferon (IFN)-γ in EAE. In addition, SA13353 attenuated the increase in IL-17-producing cells, demonstrating that SA13353 inhibited the growth of Th17 cells and development of EAE (56). Therefore, TRPV1 channel confers protection by regulating T cells in EAE.

### Pulmonary Inflammation

Pulmonary inflammation is caused by infection, physical and chemical factors, immune injury, allergy and drugs, and is mediated by a variety of inflammatory mediators such as immune cells, chemokines and cytokines. RT-PCR analysis revealed that TRPV1 was expressed in immortalized human bronchial epithelial cells, normal human bronchial/tracheal epithelial cells, and normal human small airway epithelial cells from distal airways (57). In LPS-induced lung injury, SA13353 attenuated neutrophil infiltration and enhanced the TNF-α and CINC-1 levels. In ovalbumin-induced allergic airway inflammation, SA13353 was shown to inhibit leukocyte infiltration and attenuate increase of IL-4 and IL-12p40 (58). Besides, TRPV1^+^ nociceptor sensory neurons suppressed recruitment and surveillance of neutrophils and altered lung γδ T cells through the release of the neuropeptide calcitonin gene-related peptide (CGRP) (59). In contrast, treatment with TRPV1 antagonist capsazepine or TRPV1 siRNA reduced airway hyper-responsiveness (AHR) and airway remodeling with suppressed Th2 cytokines (IL-4, IL-5 and IL-13) and epithelial cell-derived cytokines (TSLP, IL-33, and IL-25) in ovalbumin-induced chronic asthma (60). Therefore, there is a need for further studies to determine the role of TRPV1 in pneumonia.

### Inflammatory Skin Diseases

Inflammatory skin diseases refer to skin diseases caused by various internal and external infectious or non-infectious factors, which include psoriasis, atopic dermatitis, allergic contact dermatitis or irritant contact dermatitis. In the absence of tissue damage or bacterial invasion, cutaneous light stimulation triggered the release of CGRP from TRPV1^+^ neurons, which recruited IL-17a-producing γδ T cells and CD4^+^ T cells. These cells elicited a local type 17 response that augmented host defense to *C. albicans* and *S. aureus* (61). At the same time, the activated neurons could activate TRPV1^+^ neurons at an adjacent, unstimulated skin through the nerve reflex arc, which provokes the type 17 responses (61). On the other hand, psoriasis is an immune cell-mediated inflammatory skin disease, whose pathogenesis is mediated by IL-23 (62, 63). In imiquimod-induced IL-23-dependent psoriasis-like skin inflammation, TRPV1^+^ nociceptive sensory neurons were shown to interact with dermal dendritic cells to produce IL-23, thus modulating IL-17 and IL-22 production by IL23R^+^ dermal γδ T cells, which drive skin inflammation (64). Besides, atopic dermatitis (AD) is a common allergic skin disease characterized by skin barrier dysfunction, inflammation and an intense itch (65). IL-31 is an important inflammatory mediator involved in AD, which is closely associated with pruritus (66). Previous data showed that TRPV1 and TRPA1 were involved in the interaction between IL-31 and IL-31 receptor to regulate the pruritus process, which was mediated by Th2 cells in AD and skin T cell lymphoma (67). Based on the important roles played by TRPV1 in skin inflammation and pruritus, the TRPV1 channel is another potential target for skin diseases.

### Inflammatory Bowel Disease (IBD)

The occurrence of IBD is driven by chronic inflammation, which is mainly known as Crohn’s disease (CD) and ulcerative colitis (UC). Previous data showed that capsaicin, a TRPV1 agonist, attenuated severe combined immunodeficiency (SCID) T-cell transfer colitis, suggesting that the TRPV1 signaling plays a role in capsaicin-mediated attenuation of colitis (68). It was shown that TRPV1 was highly expressed in colonic nerve fibers of IBD patients (69). Luo *et al.* demonstrated high expression of TRPV1 in colonic epithelial cells and infiltrating inflammatory cells of 60 patients with active IBD (30 cases of UC and 30 cases of CD respectively), which was not associated with severity of the disease (70). Moreover, TRPV1 immunoreactive cells were robustly higher in all intestinal layers from active UC patients (71), which suggested that TRPV1 might be involved in immune cells-mediated pathogenesis of IBD. In the T-cell-mediated colitis model, TRPV1 was shown to promote T cell and intestinal inflammatory responses. Inhibition of TRPV1 in T cells by genetic factors or drugs led to reduction of the symptoms of colitis (31, 38). In addition, TRPV1 played an important role in activating mucosal macrophages and maintaining Th17 immune cells in respond to inflammatory stimuli. Overexpression of TRPV1 significantly increased the susceptibility of DSS-induced colitis and promoted DC activation and cytokine production by enhancing the activation of calcineurin/nuclear factor in activated T cell (NFATc2) signaling, and enhancing DC-mediated Th17 cell differentiation upon inflammatory stimulation (72).

In summary, the data indicated that TRPV1 might be a potential therapeutic target in the treatment of mucosal immunity and IBD.

### Osteoarthritis (OA)

Osteoarthritis (OA) is a chronic, painful and degenerative disease that affects all joint tissues and results in loss of articular cartilage. Immune cells such as macrophages and T cells in the synovium participate in stimulating and modulating inflammatory responses in OA (73). The TRPV1 mRNA and protein expression were previously detected in PBMCs from OA patients (74). TRPV1 knockout mice showed attenuated chronic phase (>6 weeks) of RA pain (75). In rat OA model, intra-articular injection of capsaicin significantly attenuated OA phenotypes, such as joint swelling, synovitis, cartilage damage, and osteophyte formation (76). Furthermore, TRPV1 alleviated OA by inhibiting M1 macrophage polarization *via* Ca^2+^/CaMKII/Nrf2 signaling pathway (76). These findings demonstrated that TRPV1 regulates various cells in OA.

## Conclusion and Future Perspectives

In this review, we analyze recent data on the expression and functions of TRPV1 in T cells and T cell-mediated inflammatory diseases. The data showed that TRPV1 is a Ca^2+^-permeable channel and mediates TCR-induced Ca^2+^ influx, leading to T cell activation and death as well as differentiation of T cell subsets. However, most of the studies only provided phenotypic observations. Therefore, data on the exact mechanisms underlying the observed phenotypic characteristics is lacking. Besides, whether TRPV1 interacts with other family members or with other channels in T cells remains unclear. In future, scientists should explore interactions between ion channels in T cells, and determine the exact cell-intrinsic roles in T cell development and in different effector T cell subsets.

Furthermore, many studies have demonstrated that TRPV1 can regulate T cell-mediated inflammation and protect the body by regulating production of T cell-related cytokines, such as TNF-α, IL-4 and IL-6. However, due to diverse expression on sensory nerves, immune cells, epithelial cells as well as the consequent activation-induced release of inflammatory mediators, the overall functions of TRPV1 in inflammatory diseases need further evaluation. These data would lay a foundation for future development of new anti-inflammatory drugs targeting TRPV1 in inflammation.

## Author Contributions

TX and MS wrote the original manuscripts. JK guided on the structure of the manuscript. CZ organized and reviewed the manuscript. CZ and TX provided the funding. All authors contributed to the article and approved the submitted version.

## Funding

This study received funding from Huai’an Natural Science Research Program (Grant No. HABL202114), the Science and Technology Development Project of Yancheng, China (YK2019108) and the Nantong University Clinical Medicine Special Project, China (2019JZ011).

## Conflict of Interest

The authors declare that the research was conducted in the absence of any commercial or financial relationships that could be construed as a potential conflict of interest.

## Publisher’s Note

All claims expressed in this article are solely those of the authors and do not necessarily represent those of their affiliated organizations, or those of the publisher, the editors and the reviewers. Any product that may be evaluated in this article, or claim that may be made by its manufacturer, is not guaranteed or endorsed by the publisher.

## References

[1] Montell C. The TRP Superfamily of Cation Channels. Sci STKE (2005) 2005(272):re3. doi: 10.1126/stke.2722005re3 15728426

[2] Montell C BL Flockerzi V. The TRP Channels, a Remarkably Functional Family. Cell (2002) 108:595–8. doi: 10.1016/s0092-8674(02)00670-0 11893331

[3] Caterina MJ SM Tominaga M Rosen TA Levine JD Julius D. The Capsaicin Receptor a Heat-Activated Ion Channel in the Pain Pathway. Nature (1997) 389(6653):816–24. doi: 10.1038/39807 9349813

[4] Talavera K Nilius B Voets T. Neuronal TRP Channels: Thermometers, Pathfinders and Life-Savers. Trends Neurosci (2008) 31(6):287–95. doi: 10.1016/j.tins.2008.03.002 18471901

[5] Liao M Cao E Julius D Cheng Y. Structure of the TRPV1 Ion Channel Determined by Electron Cryo-Microscopy. Nature (2013) 504(7478):107–12. doi: 10.1038/nature12822 PMC4078027 24305160

[6] Lishko PV Procko E Jin X Phelps CB Gaudet R. The Ankyrin Repeats of TRPV1 Bind Multiple Ligands and Modulate Channel Sensitivity. Neuron (2007) 54(6):905–18. doi: 10.1016/j.neuron.2007.05.027 17582331

[7] Phelps CB Procko E Lishko PV Wang RR Gaudet R. Insights Into the Roles of Conserved and Divergent Residues in the Ankyrin Repeats of TRPV Ion Channels. Channels (2014) 1(3):148–51. doi: 10.4161/chan.4716 18690026

[8] Garcia-Sanz N Fernandez-Carvajal A Morenilla-Palao C Planells-Cases R Fajardo-Sanchez E Fernandez-Ballester G. Identification of a Tetramerization Domain in the C Terminus of the Vanilloid Receptor. J Neurosci (2004) 24(23):5307–14. doi: 10.1523/JNEUROSCI.0202-04.2004 PMC6729306 15190102

[9] Numazaki M TT Takeuchi K Murayama N Toyooka H Tominaga M. Structural Determinant of TRPV1 Desensitization Interacts With Calmodulin. Proc Natl Acad Sci USA (2003) 100(13):8002–6. doi: 10.1073/pnas.1337252100 PMC164702 12808128

[10] Ho KW Ward N Calkins DJ. TRPV1: A Stress Response Protein in the Central Nervous System. Am J Neurodegener Dis (2012) 1(1):1–14. 22737633 PMC3560445

[11] Holzer P. The Pharmacological Challenge to Tame the Transient Receptor Potential Vanilloid-1 (TRPV1) Nocisensor. Br J Pharmacol (2008) 155(8):1145–62. doi: 10.1038/bjp.2008.351 PMC2607216 18806809

[12] Chu CJ Huang SM De Petrocellis L Bisogno T Ewing SA Miller JD. N-Oleoyldopamine, a Novel Endogenous Capsaicin-Like Lipid That Produces Hyperalgesia. J Biol Chem (2003) 278(16):13633–9. doi: 10.1074/jbc.M211231200 12569099

[13] Meotti FC Lemos de Andrade E Calixto JB. TRP Modulation by Natural Compounds. Handb Exp Pharmacol (2014) 223:1177–238. doi: 10.1007/978-3-319-05161-1_19 24961985

[14] Melnick C Kaviany M. Thermal Actuation in TRPV1: Role of Embedded Lipids and Intracellular Domains. J Theor Biol (2018) 444:38–49. doi: 10.1016/j.jtbi.2018.02.004 29425725

[15] Yao J Qin F. Interaction With Phosphoinositides Confers Adaptation Onto the TRPV1 Pain Receptor. PLoS Biol (2009) 7(2):e46. doi: 10.1371/journal.pbio.1000046 19243225 PMC3279049

[16] Prescott ED Julius D. A Modular PIP2 Binding Site as a Determinant of Capsaicin Receptor Sensitivity. Science (2003) 300(5623):1284–8. doi: 10.1126/science.1083646 12764195

[17] Ufret-Vincenty CA Klein RM Hua L Angueyra J Gordon SE. Localization of the PIP2 Sensor of TRPV1 Ion Channels. J Biol Chem (2011) 286(11):9688–98. doi: 10.1074/jbc.M110.192526 PMC3058964 21224382

[18] Cao E Cordero-Morales JF Liu B Qin F Julius D. TRPV1 Channels Are Intrinsically Heat Sensitive and Negatively Regulated by Phosphoinositide Lipids. Neuron (2013) 77(4):667–79. doi: 10.1016/j.neuron.2012.12.016 PMC3583019 23439120

[19] Senning EN Collins MD Stratiievska A Ufret-Vincenty CA Gordon SE. Regulation of TRPV1 Ion Channel by Phosphoinositide (4,5)-Bisphosphate: The Role of Membrane Asymmetry. J Biol Chem (2014) 289(16):10999–1006. doi: 10.1074/jbc.M114.553180 PMC4036241 24599956

[20] Voets T Droogmans G Wissenbach U Janssens A Flockerzi V Nilius B. The Principle of Temperature-Dependent Gating in Cold- and Heat-Sensitive TRP Channels. Nature (2004) 430(7001):748–54. doi: 10.1038/nature02732. 15306801

[21] Sappington RM Sidorova T Long DJ Calkins DJ. TRPV1: Contribution to Retinal Ganglion Cell Apoptosis and Increased Intracellular Ca2+ With Exposure to Hydrostatic Pressure. Invest Ophthalmol Vis Sci (2009) 50(2):717–28. doi: 10.1167/iovs.08-2321 PMC3549616 18952924

[22] Jeong KY Seong J. Neonatal Capsaicin Treatment in Rats Affects TRPV1-Related Noxious Heat Sensation and Circadian Body Temperature Rhythm. J Neurol Sci (2014) 341(1-2):58–63. doi: 10.1016/j.jns.2014.03.054 24746025

[23] Negri L Lattanzi R Giannini E Colucci M Margheriti F Melchiorri P. Impaired Nociception and Inflammatory Pain Sensation in Mice Lacking the Prokineticin Receptor PKR1: Focus on Interaction Between PKR1 and the Capsaicin Receptor TRPV1 in Pain Behavior. J Neurosci (2006) 26(25):6716–27. doi: 10.1523/JNEUROSCI.5403-05.2006 PMC6673825 16793879

[24] Saunders CI Kunde DA Crawford A Geraghty DP. Expression of Transient Receptor Potential Vanilloid 1 (TRPV1) and 2 (TRPV2) in Human Peripheral Blood. Mol Immunol (2007) 44(6):1429–35. doi: 10.1016/j.molimm.2006.04.027 16777226

[25] Amantini C Mosca M Lucciarini R Perfumi M Morrone S Piccoli M. Distinct Thymocyte Subsets Express the Vanilloid Receptor VR1 That Mediates Capsaicin-Induced Apoptotic Cell Death. Cell Death Differ (2004) 11(12):1342–56. doi: 10.1038/sj.cdd.4401506 15459754

[26] Farfariello V Amantini C Santoni G. Transient Receptor Potential Vanilloid 1 Activation Induces Autophagy in Thymocytes Through ROS-Regulated AMPK and Atg4C Pathways. J Leukoc Biol (2012) 92(3):421–31. doi: 10.1189/jlb.0312123 22753949

[27] Kim HS Kwon HJ Kim GE Cho MH Yoon SY Davies AJ. Attenuation of Natural Killer Cell Functions by Capsaicin Through a Direct and TRPV1-Independent Mechanism. Carcinogenesis (2014) 35(7):1652–60. doi: 10.1093/carcin/bgu091 24743513

[28] Majhi RK Sahoo SS Yadav M Pratheek BM Chattopadhyay S Goswami C. Functional Expression of TRPV Channels in T Cells and Their Implications in Immune Regulation. FEBS J (2015) 282(14):2661–81. doi: 10.1111/febs.13306 25903376

[29] Wenning AS Neblung K Strauss B Wolfs MJ Sappok A Hoth M. TRP Expression Pattern and the Functional Importance of TRPC3 in Primary Human T-Cells. Biochim Biophys Acta (2011) 1813(3):412–23. doi: 10.1016/j.bbamcr.2010.12.022 21215279

[30] Spinsanti G Zannolli R Panti C Ceccarelli I Marsili L Bachiocco V. Quantitative Real-Time PCR Detection of TRPV1-4 Gene Expression in Human Leukocytes From Healthy and Hyposensitive Subjects. Mol Pain (2008) 4:51. doi: 10.1186/1744-8069-4-51 18983665 PMC2588574

[31] Bertin S Aoki-Nonaka Y de Jong PR Nohara LL Xu H Stanwood SR. The Ion Channel TRPV1 Regulates the Activation and Proinflammatory Properties of CD4(+) T Cells. Nat Immunol (2014) 15(11):1055–63. doi: 10.1038/ni.3009 PMC4843825 25282159

[32] Samivel R Kim D Son HR Rhee YH Kim EH Kim JH. The Role of TRPV1 in the CD4+ T Cell-Mediated Inflammatory Response of Allergic Rhinitis. Oncotarget (2016) 7(1):148–60. doi: 10.18632/oncotarget.6653 PMC4807989 26700618

[33] Bertin S de Jong PR Jefferies WA Raz E. Novel Immune Function for the TRPV1 Channel in T Lymphocytes. Channels (Austin) (2014) 8(6):479–80. doi: 10.4161/19336950.2014.991640 PMC4594317 25530461

[34] Hogan PG Lewis RS Rao A. Molecular Basis of Calcium Signaling in Lymphocytes: STIM and ORAI. Annu Rev Immunol (2010) 28:491–533. doi: 10.1146/annurev.immunol.021908.132550 20307213 PMC2861828

[35] Inada H Iida T Tominaga M. Different Expression Patterns of TRP Genes in Murine B and T Lymphocytes. Biochem Biophys Res Commun (2006) 350(3):762–7. doi: 10.1016/j.bbrc.2006.09.111 17027915

[36] Zhang F Challapalli SC Smith PJ. Cannabinoid CB(1) Receptor Activation Stimulates Neurite Outgrowth and Inhibits Capsaicin-Induced Ca(2+) Influx in an *In Vitro* Model of Diabetic Neuropathy. Neuropharmacology (2009) 57(2):88–96. doi: 10.1016/j.neuropharm.2009.04.017 19501110

[37] Macho A CM Muñoz-Blanco J Gómez-Díaz C Gajate C Mollinedo F. Selective Induction of Apoptosis by Capsaicin in Transformed Cells the Role of Reactive Oxygen Species and Calcium. Cell Death Differ (1999) 6(2):155–65. doi: 10.1038/sj.cdd.4400465 10200562

[38] Bertin S Aoki-Nonaka Y Lee J de Jong PR Kim P Han T. The TRPA1 Ion Channel is Expressed in CD4+ T Cells and Restrains T-Cell-Mediated Colitis Through Inhibition of TRPV1. Gut (2017) 66(9):1584–96. doi: 10.1136/gutjnl-2015-310710 PMC5173457 27325418

[39] Szallasi A Cortright DN Blum CA Eid SR. The Vanilloid Receptor TRPV1: 10 Years From Channel Cloning to Antagonist Proof-of-Concept. Nat Rev Drug Discov (2007) 6(5):357–72. doi: 10.1038/nrd2280 17464295

[40] Evans SS Repasky EA Fisher DT. Fever and the Thermal Regulation of Immunity: The Immune System Feels the Heat. Nat Rev Immunol (2015) 15(6):335–49. doi: 10.1038/nri3843 PMC4786079 25976513

[41] Evans SS WW Bain MD Burd R Ostberg JR Repasky EA. Fever-Range Hyperthermia Dynamically Regulates Lymphocyte Delivery to High Endothelial Venules. Blood (2001) 97(9):2727–33. doi: 10.1182/blood.v97.9.2727 11313264

[42] Wang WC Goldman LM Schleider DM Appenheimer MM Subjeck JR Repasky EA. Fever-Range Hyperthermia Enhances L-Selectin-Dependent Adhesion of Lymphocytes to Vascular Endothelium. J Immunol (1998) 160(2):961–9. 9551935

[43] Lin C Zhang Y Zhang K Zheng Y Lu L Chang H. Fever Promotes T Lymphocyte Trafficking *via* a Thermal Sensory Pathway Involving Heat Shock Protein 90 and Alpha4 Integrins. Immunity (2019) 50(1):137–51.e6. doi: 10.1016/j.immuni.2018.11.013 30650373 PMC6432644

[44] Wang XY OJ Repasky EA. Effect of Fever-Like Whole-Body Hyperthermia on Lymphocyte Spectrin Distribution, Protein Kinase C Activity, and Uropod Formation. J Immunol (1999) 162(6):3378–87. 10092792

[45] Shen RN LL Young P Shidnia H Hornback NB Broxmeyer HE. Influence of Elevated Temperature on Natural Killer Cell Activity, Lymphokine-Activated Killer Cell Activity and Lectin-Dependent Cytotoxicity of Human Umbilical Cord Blood and Adult Blood Cells. Int J Radiat Oncol Biol Phys (1994) 29(4):821–6. doi: 10.1016/0360-3016(94)90571-1 8040029

[46] Wang X Ni L Wan S Zhao X Ding X Dejean A. Febrile Temperature Critically Controls the Differentiation and Pathogenicity of T Helper 17 Cells. Immunity (2020) 52(2):328–41.e5. doi: 10.1016/j.immuni.2020.01.006 32049050

[47] Gaublomme JT Yosef N Lee Y Gertner RS Yang LV Wu C. Single-Cell Genomics Unveils Critical Regulators of Th17 Cell Pathogenicity. Cell (2015) 163(6):1400–12. doi: 10.1016/j.cell.2015.11.009 PMC4671824 26607794

[48] Mace TA Zhong L Kilpatrick C Zynda E Lee CT Capitano M. Differentiation of CD8+ T Cells Into Effector Cells is Enhanced by Physiological Range Hyperthermia. J Leukoc Biol (2011) 90(5):951–62. doi: 10.1189/jlb.0511229 PMC3206471 21873456

[49] O'Sullivan D Stanczak MA Villa M Uhl FM Corrado M Klein Geltink RI. Fever Supports CD8(+) Effector T Cell Responses by Promoting Mitochondrial Translation. Proc Natl Acad Sci USA (2021) 118(25):e2023752118. doi: 10.1073/pnas.2023752118 34161266 PMC8237659

[50] Gavva NR. Body-Temperature Maintenance as the Predominant Function of the Vanilloid Receptor TRPV1. Trends Pharmacol Sci (2008) 29(11):550–7. doi: 10.1016/j.tips.2008.08.003 18805596

[51] Gavva NR Treanor JJ Garami A Fang L Surapaneni S Akrami A. Pharmacological Blockade of the Vanilloid Receptor TRPV1 Elicits Marked Hyperthermia in Humans. Pain (2008) 136(1-2):202–10. doi: 10.1016/j.pain.2008.01.024 18337008

[52] Caterina MJ Julius D. Thevanilloid Receptor a Molecular Gateway to the Pain Pathway. Annu Rev Neurosci (2001) 24:487–517. doi: 10.1146/annurev.neuro.24.1.487. 11283319

[53] Umar D Das A Gupta S Chattopadhyay S Sarkar D Mirji G. Febrile Temperature Change Modulates CD4 T Cell Differentiation *via* a TRPV Channel-Regulated Notch-Dependent Pathway. Proc Natl Acad Sci USA (2020) 117(36):22357–66. doi: 10.1073/pnas.1922683117 PMC7486768 32839313

[54] Bousounis P Bergo V Trompouki E. Inflammation, Aging and Hematopoiesis: A Complex Relationship. Cells (2021) 10(6):1386. doi: 10.3390/cells10061386 34199874 PMC8227236

[55] Paltser G Liu XJ Yantha J Winer S Tsui H Wu P. TRPV1 Gates Tissue Access and Sustains Pathogenicity in Autoimmune Encephalitis. Mol Med (2013) 19:149–59. doi: 10.2119/molmed.2012.00329 PMC3745593 23689362

[56] Tsuji F Murai M Oki K Seki I Ueda K Inoue H. Transient Receptor Potential Vanilloid 1 Agonists as Candidates for Anti-Inflammatory and Immunomodulatory Agents. Eur J Pharmacol (2010) 627(1-3):332–9. doi: 10.1016/j.ejphar.2009.10.044 19878665

[57] Agopyan N Bhatti T Yu S Simon SA. Vanilloid Receptor Activation by 2- and 10-μm Particles Induces Responses Leading to Apoptosis in Human Airway Epithelial Cells. Toxicol Appl Pharmacol (2003) 192(1):21–35. doi: 10.1016/s0041-008x(03)00259-x 14554100

[58] Tsuji F Murai M Oki K Inoue H Sasano M Tanaka H. Effects of SA13353, a Transient Receptor Potential Vanilloid 1 Agonist, on Leukocyte Infiltration in Lipopolysaccharide-Induced Acute Lung Injury and Ovalbumin-Induced Allergic Airway Inflammation. J Pharmacol Sci (2010) 112(4):487–90. doi: 10.1254/jphs.09295sc 20351486

[59] Baral P Umans BD Li L Wallrapp A Bist M Kirschbaum T. Nociceptor Sensory Neurons Suppress Neutrophil and Gammadelta T Cell Responses in Bacterial Lung Infections and Lethal Pneumonia. Nat Med (2018) 24(4):417–26. doi: 10.1038/nm.4501 PMC6263165 29505031

[60] Choi JY Lee HY Hur J Kim KH Kang JY Rhee CK. TRPV1 Blocking Alleviates Airway Inflammation and Remodeling in a Chronic Asthma Murine Model. Allergy Asthma Immunol Res (2018) 10(3):216–24. doi: 10.4168/aair.2018.10.3.216 PMC5911440 29676068

[61] Cohen JA Edwards TN Liu AW Hirai T Jones MR Wu J. Cutaneous TRPV1+ Neurons Trigger Protective Innate Type 17 Anticipatory Immunity. Cell (2019) 178(4):919–32.e14. doi: 10.1016/j.cell.2019.06.022 31353219 PMC6788801

[62] Menter A Krueger GG Paek SY Kivelevitch D Adamopoulos IE Langley RG. Interleukin-17 and Interleukin-23: A Narrative Review of Mechanisms of Action in Psoriasis and Associated Comorbidities. Dermatol Ther (Heidelb) (2021) 11(2):385–400. doi: 10.1007/s13555-021-00483-2 33512665 PMC8019008

[63] Ghoreschi K Balato A Enerbäck C Sabat R. Therapeutics Targeting the IL-23 and IL-17 Pathway in Psoriasis. Lancet (2021) 397(10275):754–66. doi: 10.1016/s0140-6736(21)00184-7 33515492

[64] Riol-Blanco L Ordovas-Montanes J Perro M Naval E Thiriot A Alvarez D. Nociceptive Sensory Neurons Drive Interleukin-23-Mediated Psoriasiform Skin Inflammation. Nature (2014) 510(7503):157–61. doi: 10.1038/nature13199 PMC4127885 24759321

[65] Nakahara T Kido-Nakahara M Tsuji G Furue M. Basics and Recent Advances in the Pathophysiology of Atopic Dermatitis. J Dermatol (2021) 48(2):130–9. doi: 10.1111/1346-8138.15664 33118662

[66] Imai Y. Interleukin-33 in Atopic Dermatitis. J Dermatol Sci (2019) 96(1):2–7. doi: 10.1016/j.jdermsci.2019.08.006 31455506

[67] Cevikbas F Wang X Akiyama T Kempkes C Savinko T Antal A. A Sensory Neuron-Expressed IL-31 Receptor Mediates T Helper Cell-Dependent Itch: Involvement of TRPV1 and TRPA1. J Allergy Clin Immunol (2014) 133(2):448–60. doi: 10.1016/j.jaci.2013.10.048 PMC3960328 24373353

[68] Belmaati MS Diemer S Hvarness T Baumann K Pedersen AE Christensen RE. Antiproliferative Effects of TRPV1 Ligands on Nonspecific and Enteroantigen-Specific T Cells From Wild-Type and Trpv1 KO Mice. Inflammation Bowel Dis (2014) 20(6):1004–14. doi: 10.1097/MIB.0000000000000039 24788222

[69] Yiangou Y Facer P Dyer NH Chan CL Knowles C Williams NS. Vanilloid Receptor 1 Immunoreactivity in Inflamed Human Bowel. Lancet (2001) 357(9265):1338–9. doi: 10.1016/s0140-6736(00)04503-7 11343743

[70] Luo C Wang Z Mu J Zhu M Zhen Y Zhang H. Upregulation of the Transient Receptor Potential Vanilloid 1 in Colonic Epithelium of Patients With Active Inflammatory Bowel Disease. Int J Clin Exp Pathol (2017) 10(11):11335–44. PMC6965867 31966488

[71] Toledo-Maurino JJ Furuzawa-Carballeda J Villeda-Ramirez MA Fonseca-Camarillo G Meza-Guillen D Barreto-Zuniga R. The Transient Receptor Potential Vanilloid 1 Is Associated With Active Inflammation in Ulcerative Colitis. Mediators Inflamm (2018) 2018:6570371. doi: 10.1155/2018/6570371 30150894 PMC6087567

[72] Duo L Wu T Ke Z Hu L Wang C Teng G. Gain of Function of Ion Channel TRPV1 Exacerbates Experimental Colitis by Promoting Dendritic Cell Activation. Mol Ther Nucleic Acids (2020) 22:924–36. doi: 10.1016/j.omtn.2020.10.006 PMC7666365 33251043

[73] Woodell-May JE Sommerfeld SD. Role of Inflammation and the Immune System in the Progression of Osteoarthritis. J Orthop Res (2020) 38(2):253–7. doi: 10.1002/jor.24457 31469192

[74] Engler A Aeschlimann A Simmen BR Michel BA Gay RE Gay S. Expression of Transient Receptor Potential Vanilloid 1 (TRPV1) in Synovial Fibroblasts From Patients With Osteoarthritis and Rheumatoid Arthritis. Biochem Biophys Res Commun (2007) 359(4):884–8. doi: 10.1016/j.bbrc.2007.05.178 17560936

[75] Hsieh WS Kung CC Huang SL Lin SC Sun WH. TDAG8, TRPV1, and ASIC3 Involved in Establishing Hyperalgesic Priming in Experimental Rheumatoid Arthritis. Sci Rep (2017) 7(1):8870. doi: 10.1038/s41598-017-09200-6 28827659 PMC5566336

[76] Lv Z Xu X Sun Z Yang YX Guo H Li J. TRPV1 Alleviates Osteoarthritis by Inhibiting M1 Macrophage Polarization *via* Ca(2+)/CaMKII/Nrf2 Signaling Pathway. Cell Death Dis (2021) 12(6):504. doi: 10.1038/s41419-021-03792-8 34006826 PMC8131608

